# Acceptability and Feasibility of Repeated Mucosal Specimen Collection in Clinical Trial Participants in Kenya

**DOI:** 10.1371/journal.pone.0110228

**Published:** 2014-10-31

**Authors:** Gloria Omosa-Manyonyi, Harriet Park, Gaudensia Mutua, Bashir Farah, Philip J. Bergin, Dagna Laufer, Jennifer Lehrman, Kundai Chinyenze, Burc Barin, Pat Fast, Jill Gilmour, Omu Anzala

**Affiliations:** 1 Kenya AIDS Vaccine Initiative Institute of Clinical Research, University of Nairobi, Nairobi, Kenya; 2 International AIDS Vaccine Initiative, New York, New York, United States of America; 3 IAVI Human Immunology Laboratory, Imperial College London, London, United Kingdom; 4 EMMES Corporation, Rockville, Maryland, United States of America; University of Cape Town, South Africa

## Abstract

**Background:**

Mucosal specimens are essential to evaluate compartmentalized immune responses to HIV vaccine candidates and other mucosally targeted investigational products. We studied the acceptability and feasibility of repeated mucosal sampling in East African clinical trial participants at low risk of HIV and other sexually transmitted infections.

**Methods and Findings:**

The Kenya AIDS Vaccine Initiative (KAVI) enrolled participants into three Phase 1 trials of preventive HIV candidate vaccines in 2011–2012 at two clinical research centers in Nairobi. After informed consent to a mucosal sub-study, participants were asked to undergo collection of mucosal secretions (saliva, oral fluids, semen, cervico-vaginal and rectal), but could opt out of any collection at any visit. Specimens were collected at baseline and two additional time points. A tolerability questionnaire was administered at the final sub-study visit. Of 105 trial participants, 27 of 34 women (79%) and 62 of 71 men (87%) enrolled in the mucosal sub-study. Nearly all sub-study participants gave saliva and oral fluids at all visits. Semen was collected from about half the participating men (47–48%) at all visits. Cervico-vaginal secretions were collected by Softcup from about two thirds of women (63%) at baseline, increasing to 78% at the following visits, with similar numbers for cervical secretion collection by Merocel sponge; about half of women (52%) gave cervico-vaginal samples at all visits. Rectal secretions were collected with Merocel sponge from about a quarter of both men and women (24%) at all 3 visits, with 16% of men and 19% of women giving rectal samples at all visits.

**Conclusions:**

Repeated mucosal sampling in clinical trial participants in Kenya is feasible, with a good proportion of participants consenting to most sampling methods with the exception of rectal samples. Experienced staff members of both sexes and trained counselors with standardized messaging may improve acceptance of rectal sampling.

## Introduction

In sub-Saharan Africa and other low and middle income countries that bear the brunt of the pandemic, the dominant route of transmission of HIV-1 is across the genital mucosa during sexual intercourse. Immune responses, both humoral and cellular, have been identified at mucosal surfaces and may be protective [1], [2]. As new vaccine candidates with the potential of inducing a compartmentalized mucosal response have become available (e.g., Sendai virus vector, now in trial in East Africa; ClinicalTrials.gov Identifier NCT01705990), the ability to induce protective responses in the mucosa is a key opportunity to control or even halt HIV infection in its early stages [3].

Mucosal sampling in HIV preventive trials is becoming more common, but much of the work is being conducted in North America or Europe [4]–[8]. Sub-Saharan Africa, the region with the highest HIV prevalence and incidence, will be a site of future efficacy trials and would be the region to benefit the most from an efficacious HIV vaccine. Additionally, there is a high likelihood of population-specific differences in mucosal immune responses, due to genetic factors and effects from endemic infections and environmental factors. It is therefore important to build capacity to collect, process, and analyze mucosal specimens at sub-Saharan African clinical trial centers. A large body of mucosal work in HIV-exposed but uninfected populations has already been done in Nairobi [1], [2], [9].

The International AIDS Vaccine Initiative (IAVI) and the Kenya AIDS Vaccine Initiative Institute of Clinical Research (KAVI-ICR) of the University of Nairobi have been working together to adapt existing mucosal sample collection and analysis methods and test new ones in preparation for HIV vaccine clinical trials. The current study was attached to three IAVI-sponsored Phase 1 HIV vaccine trials in Nairobi as an optional sub-study to assess acceptability of repeated mucosal sampling and the nature of vaccine-induced mucosal HIV-1-specific immune responses. This paper reviews the acceptability of a wide range of mucosal sampling methods including rectal, oral, cervico-vaginal secretions and semen, taken at three time points within the main vaccine trial schedule.

## Methods

This mucosal sub-study recruited participants who had enrolled in one of three IAVI-sponsored HIV preventive vaccine trials conducted either at the main KAVI clinical research center at the Kenyatta National Hospital (KNH) complex or at KAVI's research unit in Kangemi, on the outskirts of Nairobi. The mucosal and vaccine trial protocols were approved by the Kenyatta National Hospital-University of Nairobi Ethical Review Committee. After written informed consent and enrollment into the main vaccine trial, participants underwent the informed consent process and signed a separate consent form for the mucosal study. Participants who did not initially consent to the mucosal sub-study could enroll at any time before the final study visit.

### Study Participants

Eligibility criteria for the three vaccine trials included being healthy, at low risk for HIV and between the ages of 18 and 50 (18–40 years for one trial, B002). All participants were advised to use condoms. In addition, a long-lasting non-barrier method of contraception, such as Depo-Provera, Norplant, intra-uterine device (IUD) or tubal ligation was required of all female participants of child-bearing potential (oral contraceptives were not allowed). Female participants with an IUD were excluded from cervico-vaginal sampling due to a risk of the IUD being dislodged by the Softcup [10].

### Vaccine Trials and Study Schedule

Participants were drawn from the following Phase 1 vaccine trials: IAVI B002 (ClinicalTrials.gov Identifier NCT01264445), B003 (NCT01215149), and B004 (NCT01496989), conducted at sites in Eastern Africa, South Africa, and the USA, with the mucosal sub-study conducted at the Kenyan sites only. In Kenya, trials B002 and B003 were conducted in 2011–2012 at KAVI-KNH and KAVI-Kangemi, respectively, and B004 was conducted in 2012–2013 at KAVI-Kangemi. Mucosal specimens were collected at three time points for each trial, with the timing dependent on trial design: in B002 and B003, sampling was at baseline, one month after the final vaccination and at the next vaccine trial visit; in B004 sampling was at baseline, one month after the prime and one month after the boost.

### Study Procedures

Participants were free to opt out of any collection at any time or to provide samples they had previously refused. Reasons for refusing any sample collection were recorded at each visit. A questionnaire was administered at the final mucosal study visit, asking participants the main reason they agreed to provide mucosal specimens, what mucosal specimens they would agree to in future studies, and any general suggestions for making the procedures more tolerable. Questions were open-ended. If responses fit with a pre-determined list of responses, answers were coded accordingly. If responses did not fit with one of the pre-set answers, they were recorded verbatim. Up to two reasons for refusal were collected at each visit for each sample type not given.

Saliva was collected by placing a Salimetrics Oral Swab (Salimetrics LLC, State College, PA, USA) against the parotid duct for 5 minutes. Oral fluid (transudate) was collected by allowing fluids to pool in the mouth then passed into a Falcon tube [11]. Participants were instructed not to eat or drink anything but water for 2 hours prior to saliva and oral fluid collection.

In female participants, the Instead Softcup (Evofem Inc., San Diego, CA, USA), was inserted by the clinician and kept in place for 5 minutes (10 minutes for B004) to collect cervico-vaginal secretions. The cervix was then accessed with a disposable speculum and two pre-moistened Merocel sponges (Medtronic, Minneapolis, MN, USA) were placed against the cervical mucosa for 5 minutes each, serially. The Softcup and Merocel sponge have been used to collect cervico-vaginal secretions in other research studies [12], [13]. Cervico-vaginal collection was not performed during menstruation. If possible, samples were taken approximately 2 days after bleeding ended, except baseline samples, which were considered missed if the participant was menstruating on the day of vaccination. Male participants provided semen specimens, by masturbation, into a universal container.

Rectal secretions were collected from both male and female participants by accessing the rectal mucosa through a disposable clinician-inserted proctoscope. Rectal secretions were collected using two pre-moistened Merocel sponges placed against the rectal mucosa for 5 minutes each, serially.

B002 and B003 participants were reimbursed a set amount at the end of each visit, regardless of the actual collections performed. In B004, the reimbursement structure was changed so that participants were given a set amount per sample type in order to reimburse participants for the significant additional time involved in providing all specimen types as opposed to just one.

Humoral responses were assessed by anti-HIV specific IgG and IgA ELISAs on frozen samples. Results will be published separately.

### Statistical Methods

Participants' overall acceptance of a mucosal sampling method was calculated as the proportion of participants who provided any specimen for that sampling method during the study. 95% confidence intervals for the observed proportions were estimated using exact (Clopper-Pearson) binomial method in PASS 2008 (NCSS, Kaysville, UT).

Due to limited sample size, the statistical comparisons were primarily exploratory and were conducted for evaluation of any observed site, trial or gender differences in acceptability of mucosal sampling methods. Comparisons of categorical and continuous factors were conducted using the Fisher's exact test and Wilcoxon rank-sum test, respectively. A two-sided p-value of less than 0.05 was considered to be statistically significant. Statistical analyses were performed using SAS version 9.3 (SAS Institute Inc., Cary, NC).

## Results

### Participant Characteristics at Enrollment

Male participants in the vaccine trials outnumbered female participants 2∶1 overall and this disproportion was also reflected in the mucosal sub-study. 27/34 (79%) females and 62/71 (87%) males consented and enrolled in the mucosal sub-study (p = 0.11, Table 1). Participant ages ranged from 18 to 46 years. B002 participants were significantly younger (p<0.0001) than participants in the other two trials due to the stricter age criteria specified in the protocol. Most female participants in all three trials were on an injectable hormonal contraceptive (Depo-Provera), although a few used Norplant or had a tubal ligation. Three female participants had an IUD and were therefore excluded from cervico-vaginal collection.

**Table 1:** Participant characteristics at enrollment.

Vaccine Trial	B002		B003		B004		Total	
	Female	Male	Female	Male	Female	Male	Female	Male
# Participants Enrolled in Vaccine Trial	11	29	13	27	10	15	34	71
# Participants Enrolled in Mucosal Substudy (%)	9 (82)	28 (97)	8 (62)	20 (74)	10 (100)	14 (93)	27 (79)	62 (87)
Median Age Enrolled in Mucosal Substudy (range)	23 (18–37)	23 (18–33)	27 (22–43)	25 (19–41)	28 (25–46)	28 (21–45)	27 (18–46)	24 (18–45)

Of 16 vaccine trial participants that were not enrolled in the mucosal study, seven participants cited discomfort or fear of the sampling methods, two refused without giving a specific reason, for two participants the consent form was not yet available in the local language, and one participant each reported not wanting more procedures, lack of time or parental advice against joining. One participant was excluded for vertebral and pelvic bone deformities and one participant was not enrolled because vaccinations were discontinued following an adverse event not related to vaccination. Participants who declined participation in the mucosal study did not differ significantly in gender and age characteristics from those who participated.

### Acceptability of Sample Collection

**Figure 1:**
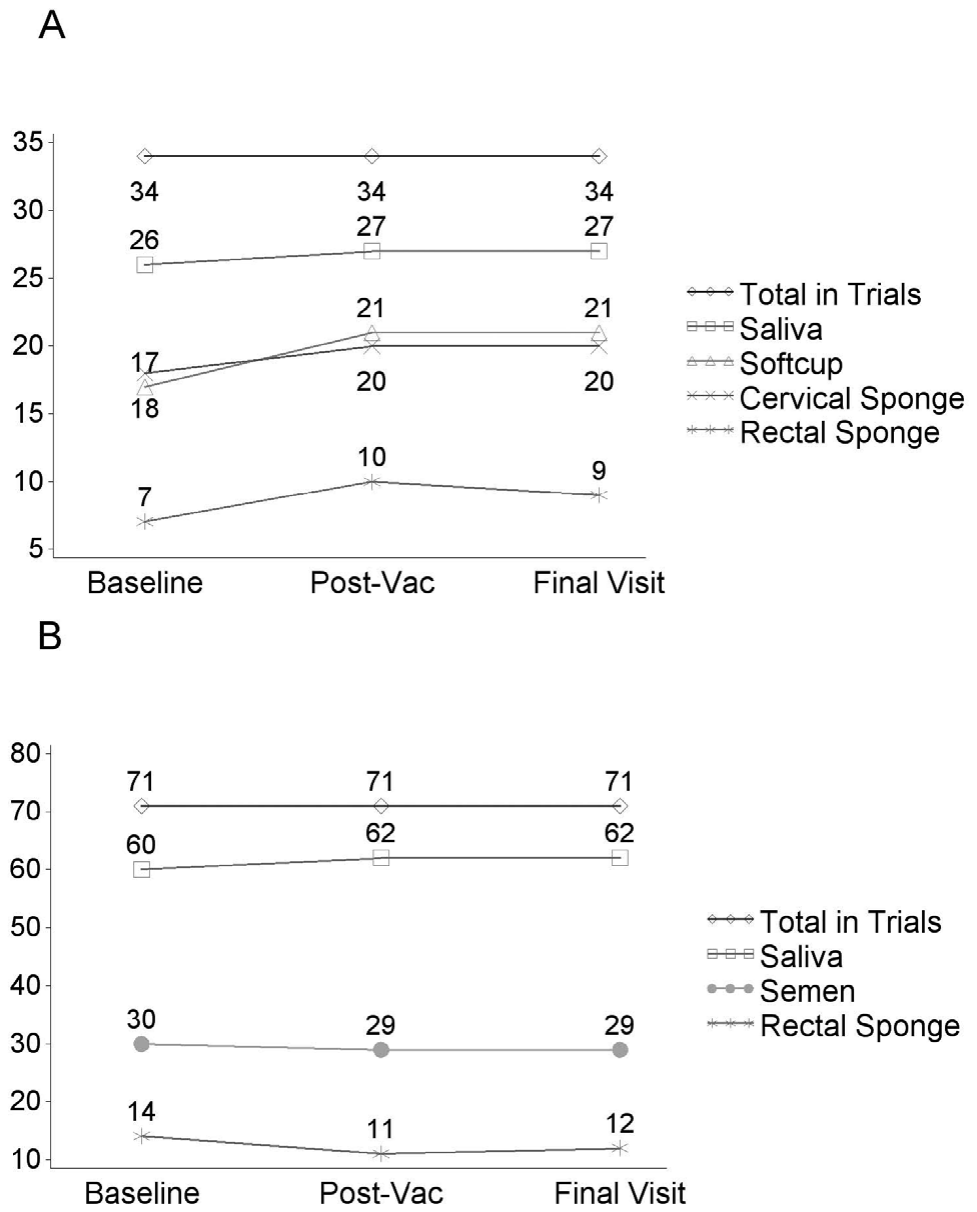
Total number of participants undergoing mucosal collection by gender. A. Total number of female participants in the three trials combined and the number providing each type of specimen at the three time points. B. Total combined number of male participants and the number providing specimens at each time point.

Saliva and oral fluids, the least invasive samples, were collected from all participants in the mucosal sub-study at nearly all visits, while rectal sample collection was the least likely to be accepted (Figure 1). Based on overall acceptance and corresponding 95% confidence intervals, female participants agreed to both types of cervico-vaginal sampling more readily than rectal sampling (Table 2). Similarly, male participants were more likely to provide semen than rectal samples.

**Table 2:** Number (%) of participants providing a total of 0, 1, 2 or 3 specimens in mucosal substudy and overall acceptance of mucosal sampling by gender*.

	Female (N = 27)					Male (N = 62)				
# Specimens	0	1	2	3	Overall acceptance (95%CI)†	0	1	2	3	Overall acceptance (95%CI)†
Total Saliva	0 (0)	0(0)	1 (4)	26 (96)	100 (87–100)	0 (0)	0 (0)	2 (3)	60 (97)	100 (94–100)
Total Semen	N/A	N/A	N/A	N/A	N/A	30 (48)	3 (5)	2 (3)	27 (44)	52 (39–65)
Total Softcup	4 (15)	1 (4)	8 (30)	14 (52)	85 (66–96)	N/A	N/A	N/A	N/A	N/A
Total Cervical Sponge	4 (15)	2 (7)	7 (26)	14 (52)	85 (66–96)	N/A	N/A	N/A	N/A	N/A
Total Rectal Sponge	16 (59)	1 (4)	5 (19)	5 (19)	41 (22–61)	46 (74)	5 (8)	1 (2)	10 (16)	26 (16–39)

*No drop-outs over time were observed in the study.

† Calculated as the percentage of participants who provided any specimen for that sampling method during the study; CI: Confidence Interval.

Cervico-vaginal sampling by Softcup and Merocel sponge was well-accepted and tolerated by the majority of women. Of 27 female participants, 18 (67%) had cervico-vaginal samples collected with Merocel sponge at baseline and 17 (63%) also had cervico-vaginal samples collected with Softcup (Table 3). Consent for cervico-vaginal sampling remained consistent across the two follow-up visits. Most missed samples were attributable to menstruation or IUD; only three participants refused cervico-vaginal sampling because of physical or emotional discomfort (data not shown). Participants in B004 were given the choice of self-inserting the Softcup or having a clinician place the device. All participants chose to have a clinician insert the Softcup.

**Table 3:** Number of participants providing specimens at each visit.

	# Participants Giving Samples Baseline (%)		# Participants Giving Samples Second Visit (%)		# Participants Giving Samples Final Visit (%)	
	Female	Male	Female	Male	Female	Male
B002 Saliva	8 (89)	26 (93)	9 (100)	28 (100)	9 (100)	28 (100)
B002 Semen	N/A	9 (32)	N/A	8 (29)	N/A	7 (25)
B002 Softcup	6 (67)	N/A	7 (78)	N/A	6 (67)	N/A
B002 Cervical Sponge	6 (67)	N/A	7 (78)	N/A	6 (67)	N/A
B002 Rectal Sponge	2 (22)	3 (11)	2 (22)	3 (11)	1 (11)	3 (11)
B003 Saliva	8 (100)	20 (100)	8 (100)	20 (100)	8 (100)	20 (100)
B003 Semen	N/A	12 (60)	N/A	12 (60)	N/A	13 (65)
B003 Softcup	3* (38)	N/A	6 (75)	N/A	7 (88)	N/A
B003 Cervical Sponge	4* (50)	N/A	5 (63)	N/A	6 (75)	N/A
B003 Rectal Sponge	2 (25)	6 (30)	1 (13)	2 (10)	1 (13)	2 (10)
B004 Saliva	10 (100)	14 (100)	10 (100)	14 (100)	10 (100)	14 (100)
B004 Semen	N/A	9 (64)	N/A	9 (64)	N/A	9 (64)
B004 Softcup	8 (80)	N/A	8 (80)	N/A	8 (80)	N/A
B004 Cervical Sponge	8 (80)	N/A	8 (80)	N/A	8 (80)	N/A
B004 Rectal Sponge	3 (30)	5 (36)	7 (70)	6 (43)	7 (70)	7 (50)
Total Saliva	26 (96)	60 (97)	27 (100)	62 (100)	27 (100)	62 (100)
Total Semen	N/A	30 (48)	N/A	29 (47)	N/A	29 (47)
Total Softcup	17 (63)	N/A	21 (78)	N/A	21 (78)	N/A
Total Cervical Sponge	18 (67)	N/A	20 (74)	N/A	20 (74)	N/A
Total Rectal Sponge	7 (26)	14 (23)	10 (37)	11 (18)	9 (33)	12 (19)

*Two women missed baseline cervico-vaginal samples due to menstruation.

Overall, semen was provided by 30 out of 62 (48%) male participants across all studies at baseline, with a similar percentage (29/62, 47%) at the second and third visits (Table 3). There was a significant difference in the proportion of male participants providing any semen specimen in B002, conducted at the KNH clinic (32%) and in B003 and B004, conducted at the Kangemi clinic (68%, p = 0.01). Among participants who did not provide semen specimens, approximately half cited embarrassment/emotional discomfort as the main reason. Another 8 (23%) were specifically uncomfortable about masturbating at the clinic (Table 4).

**Table 4:** Main reason specimen not collected (reported at first refusal) – number of participants (% of refusals).

Specimens	Semen	Softcup	Cervical Sponge	Rectal Sponge
Too invasive				6 (8)
Physical discomfort or pain		2 (15)	1 (8)	19 (26)
Embarrassment/emotional discomfort	17 (49)	2 (15)	2 (15)	34 (46)
Partner/family disapproval	1 (3)			
Uncomfortable masturbating in the clinic	8 (23)			
Concern about inability to provide required specimen on demand	1 (3)			
Procedures too time consuming	1 (3)			1 (1)
Menstruating		4 (31)	4 (31)	1 (1)
Clinician decision that collection is contraindicated		3 (23)	2 (15)	
Discomfort with the clinician				1 (1)
Difficulty masturbating	4 (11)			
Religious reasons	2 (6)			5 (7)
Being in a hurry	1 (3)			1 (1)
Pregnancy			1 (8)	1 (1)
Site decision		1 (8)	1 (8)	2 (3)
Being scared about the procedure			1 (8)	
Not wanting rectal exam				2 (3)
Procedure being unnatural				1 (1)
Not reported		1 (8)	1 (8)	

Rectal secretions were collected with Merocel sponge from 21/89 (24%) male and female participants at each of the three visits, however the male to female ratio differed slightly with each visit (Table 3). Overall, there was no significant difference in the proportion of participants providing any rectal sponge specimens between females (41%) and males (26%) (Table 2; p = 0.21). The proportion of participants who provided any rectal specimen was similar for B002 and B003 (16% combined), but significantly larger for B004 participants (56%) (data not shown; p = 0.0002). A slight downward trend over time in B002 and B003 was reversed in B004, with more participants agreeing to rectal sampling at the second and third visits compared to baseline (Table 3).

When asked at the final mucosal study visit the reason for agreeing to provide specimens, contribution to HIV research was the primary reason given (50–64% for various specimen types), finding out more about one's health ranked second (20–32%), belief that the samples were a requirement ranked third (4–12%), belief that the study would help them access more health care ranked fourth (4–5%), and “easiness of giving” was specified as another reason for providing specimens (3–9%, with the highest rate being for saliva). When asked for suggestions to improve the mucosal sampling experience, 32 out of 89 (36.0%) participants said they had no problems or issues with the sampling methods in this study and 28/89 (31.5%) had no comments. Suggestions to improve semen sampling included quieter rooms, provision of pornographic materials (not used in this study due to Kenyan anti-pornography law) or allowing partners to assist the participant. Rectal sampling was another area of focus, with suggestions to use a smaller proctoscope or to find a method without the need for a proctoscope. Suggestions are summarized in Table S1.

## Discussion

This study aimed to assess the acceptability of various mucosal sampling methods in healthy, adult, HIV-uninfected Kenyan clinical trial participants over the course of three visits. While the study was done within the context of HIV vaccine trials, the results apply to any type of study requiring mucosal sampling. The acceptability, as measured by proportion of samples collected, varied by sample type. Saliva was easily accessed and given by all participants. Cervico-vaginal and semen sample collection rates were not as high as saliva but many participants consented to the procedures and those that did remained consistent across visits. Rectal sampling was the least acceptable, with significant variance between study sites.

Cervico-vaginal sample collection had high acceptability and tolerability, but many samples were missed because of menstruation at baseline, or because the participants had IUDs. Although the IUD exclusion was connected to the Softcup, the protocol was written in such a way that IUDs excluded participants from all cervico-vaginal collection. Otherwise, the rate of cervico-vaginal sponge collection may have been higher. The three women who refused cervico-vaginal sampling cited physical and emotional discomfort as their reasons.

The Softcup is a well-accepted device for collection of menstrual fluid by self-insertion [14] and has been used to obtain self-collected cervico-vaginal specimens in clinical trials [15]. It was therefore chosen for this study to collect undiluted samples with the hypothesis that a self-inserted method would improve acceptability. Yet when participants were given the option of self-insertion, all chose clinician insertion of the Softcup. Perhaps non-familiarity with the Softcup device in this population contributed to this phenomenon; a survey by Rositch et al reported 82% acceptability of self-sampling for pap-smear screening among women in Nairobi [16]. Larger studies of women from different cultures to further understand their preferred methods for collection of genital mucosal samples are needed. The aversion to self-inserting the Softcup in this population would seem to abrogate its perceived benefit. It was thought that using a dry Merocel sponge might disrupt the mucosal epithelium, however the pre-wet sponge results in a diluted sample. The use of dry sponges and other cervico-vaginal collection methods should be further explored.

Semen samples were more acceptable at the Kangemi center than at the KNH center. A possible contributor to the difference was that private rooms were more readily available at Kangemi than KNH at the time of the study. Additionally, all the clinic personnel at KNH were female as opposed to Kangemi, which had one male nurse and a male clinical officer. Some participants declined or were unsuccessful in providing a semen sample the same day as other samples but did so without any problems on a return visit when fewer specimens were collected. This suggests that multiple complex specimen collections at a single visit may be less feasible.

Collection of rectal secretion with Merocel sponge was the most challenging. Differences between the studies could be attributable to a number of factors. One possible explanation is that the staff at one site were relatively inexperienced with rectal sampling initially, but became more adept by the time the next study was conducted. It is conceivable that word spread amongst the participants in the last study that the procedure was tolerable. Additionally, the staff's increased familiarity and comfort with rectal sampling may have come through in the counseling process, increasing the participants' willingness to consent.

Another contributing factor may have been the different reimbursement schemes. B002 and B003 participants received the same reimbursement whether they gave rectal samples or not. B004 participants received additional reimbursement for each sample type they agreed to. Although rectal specimen collection was significantly greater in B004 compared to B002 and B003, no one, including B004 participants, cited money as the reason they gave any particular sample. More than half reported that they gave samples for altruistic reasons. Without a comparison group at the same site at the same time, it is difficult to draw any conclusions about the impact of the different reimbursement schemes.

It is discouraging that despite an in-depth information session(s) and consent process, 4–12% of the participants, depending on sample type, gave the sample because they thought it was required. In the clinical trials that this mucosal sub-study was nested, blood draws were a requirement; it is therefore possible that participants may have assumed that the same requirement applied to the mucosal samples, especially since specimen sampling for the two studies coincided. It is also likely that participants understood they had a choice about which samples to provide when they first entered the study, but may have thought that once they agreed to something it was required for the remaining visits. We recommend that consent forms include a section where the participant can indicate which sampling methods they consent to. Since the consent form is signed at the beginning of the study, we also recommend verbal confirmation of which samples the participant is agreeing to at each visit. This should be documented in writing in clinic notes or other source documents. KAVI is currently conducting a clinical trial with these added precautions. We plan to compare participants' reasons for agreeing to provide specimens across these studies and see if this additional step has improved understanding of the study requirements.

### Study Limitations

The demographic data collected in the three vaccine trials were limited to age, gender, and race/ethnicity. As a result, other information such as marital status, education, parity for women and socio-economic differences could not be examined. The study participants were selected for their low risk of HIV infection, and their knowledge, attitudes and flexibility may differ from people who are at higher risk, particularly sex workers.

The mucosal sampling time points in each trial were dependent on the vaccination schedules, which varied between protocols. The length of the gap between the second and third visits did not appear to have affected compliance.

The informed consent process was similar between the two sites, but scripts were not used to explain the procedures. Individual differences between counselors could have affected the likelihood of enrollment. Without a strictly standardized method of explaining the procedures, it is possible the consent process had an impact on the initial acceptability of the various sample types. This is an area for further development and research.

## Conclusions

Repeated mucosal sampling including saliva, oral fluids, semen, cervico-vaginal and rectal specimens in healthy, adult, HIV-uninfected clinical trial participants in Kenya is feasible. Participants consented to most specimen collection methods with the exception of rectal sampling. Given the high HIV incidence demonstrated in MSM populations in Africa [17], rectal mucosal sampling should not be dismissed because of its challenges. Experienced staff members that include both men and women, well-trained counselors and standardized language during the informed consent process may improve acceptability of rectal and other sampling.

## Supporting Information

Table S1 **Participant suggestions for improving mucosal sampling experience, n = 89.**
(DOCX)
