# Supplementary material for: Acceptability and Feasibility of Repeated Mucosal Specimen Collection in Clinical Trial Participants in Kenya
Source: PLoS One. 2014 Oct 31;9(10):e110228. doi: 10.1371/journal.pone.0110228 (PMC4215886; doi:10.1371/journal.pone.0110228)
Supplement: Table S1 — Participant suggestions for improving mucosal sampling experience, n = 89. (DOCX) [file pone.0110228.s001.docx]

**Table S1: Participant suggestions for improving mucosal sampling experience, n=89**

| **Suggestions** | **Number*** |
| --- | --- |
| **Semen** | |
| Provide pornographic materials or allow partner | 6 |
| Allow semen collection at home | 3 |
| Provide quieter room for collection | 3 |
| Provide larger container to avoid spillage | 1 |
| **Rectal** | |
| Use a smaller proctoscope | 4 |
| Find a way to sample without the proctoscope | 3 |
| Find a self-collected method of rectal sampling | 2 |
| Associates rectal sampling with homosexuality | 2 |
| Has no suggestion, just not comfortable with any rectal sampling | 1 |
| **Other Comments** | |
| Give more time for consent process | 1 |
| Have the same doctor follow up at all study visits | 1 |
| Just do saliva collection; the others are too involving | 1 |
| Use straw for oral fluid collection | 1 |
| Remind participants when a mucosal collection visit is due (participant misplaced visit calendar) | 1 |
| No problem with methods used in this study | 32 |
| **No Comment** | 28 |

*Out of 89 participants, 88 gave up to one suggestion. One participant gave two suggestions.
